# USING AN MBSR MOBILE APP FOR MENTAL HEALTH: EXPERIENCES OF OLDER ADULTS IN UNDERSERVED COMMUNITIES

**DOI:** 10.1093/geroni/igae098.3443

**Published:** 2024-12-31

**Authors:** Cristina de Rosa, Yu-Ping Chang

**Affiliations:** University at Buffalo, SUNY, Buffalo, New York, United States; University at Buffalo, SUNY, Buffalo, New York, United States

## Abstract

Older adults living in underserved and racial/ethnic minority communities during the COVID-19 pandemic and beyond faced challenges regarding access to mental health care, compounded by additional vulnerability to disease. Technological solutions such as mobile applications provide great opportunities to address mental health concerns. This qualitative, descriptive analysis explores experiences of participating in an interactive and culturally-tailored mindfulness-based stress reduction (MBSR) mobile application intervention for mental health among older adults from an underserved, primarily African American community. Individual semi-structured interviews were conducted with participants who completed an 8-week MBSR mobile app intervention. Interviews were transcribed verbatim and analyzed using Braun and Clarke’s reflexive thematic analysis. A total of 17 participants were included in the analysis. Participants were ages 60-84 (M = 65.76, SD = 6.02); 94.1% were female; 82.4% reported their race/ethnicity as Black or African American. Four themes were identified in our preliminary analysis: app technology facilitated an openness to mindfulness and deconstructed barriers to access, enabled participants to come into a greater awareness of self and surroundings, allowed on-demand practices particularly during prime opportunities and stressful situations, and encouraged personal habits for maintaining and revisiting calm and relaxing states. Findings suggest feasibility and acceptability of a mobile app-delivered MBSR intervention addressing mental health needs for older adults, as well as satisfaction with app content, delivery, and support. Innovative technologies such as mobile apps have immense potential to bridge gaps in care and should be further evaluated for effectiveness and possible implementation to ameliorate mental health symptoms in underserved communities.

